# Serum glial cell line-derived neurotrophic factor (GDNF) a potential biomarker of executive function in Parkinson’s disease

**DOI:** 10.3389/fnins.2023.1136499

**Published:** 2023-02-23

**Authors:** Shu-Yan Tong, Rui-Wen Wang, Qian Li, Yi Liu, Xiao-Yan Yao, De-Qin Geng, Dian-Shuai Gao, Chao Ren

**Affiliations:** ^1^Department of Neurology, The Second Affiliated Hospital of Xuzhou Medical University, General Hospital of Xuzhou Mining Group, Xuzhou, Jiangsu, China; ^2^Department of Anesthesiology, Yantai Yuhuangding Hospital, Qingdao University, Yantai, Shandong, China; ^3^Department of Scientific Research, Yantai Yuhuangding Hospital, Qingdao University, Yantai, Shandong, China; ^4^Department of Cell Biology and Neurobiology, Xuzhou Key Laboratory of Neurobiology, Xuzhou Medical University, Xuzhou, Jiangsu, China; ^5^Department of Neurology, Yantai Yuhuangding Hospital, Qingdao University, Yantai, Shandong, China; ^6^Department of Neurology, Affiliated Hospital of Xuzhou Medical University, Xuzhou, Jiangsu, China; ^7^Shandong Provincial Innovation and Practice Base for Postdoctors, Yantai Yuhuangding Hospital, Yantai, Shandong, China; ^8^Department of Otorhinolaryngology, Head and Neck Surgery, Yantai Yuhuangding Hospital, Qingdao University, Yantai, Shandong, China

**Keywords:** Parkinson’s disease, executive function, glial cell line-derived neurotrophic factor, homovanillic acid, neuropsychological test

## Abstract

**Objective:**

Evidence shows that the impairment of executive function (EF) is mainly attributed to the degeneration of frontal-striatal dopamine pathway. Glial cell line-derived neurotrophic factor (GDNF), as the strongest protective neurotrophic factor for dopaminergic neurons (DANs), may play a role in EF to some extent. This study mainly explored the correlation between serum GDNF concentration and EF performance in Parkinson’s disease (PD).

**Methods:**

This study recruited 45 healthy volunteers (health control, HC) and 105 PD patients, including 44 with mild cognitive impairment (PD-MCI), 20 with dementia (PD-D), and 20 with normal cognitive function (PD-N). Neuropsychological tests were performed to evaluate EF (working memory, inhibitory control, and cognitive flexibility), attention, language, memory, and visuospatial function. All subjects were tested for serum GDNF and homovanillic acid (HVA) levels by ELISA and LC-ESI-MS/MS, respectively.

**Results:**

PD-MCI patients showed impairments in the trail making test (TMT) A (TMT-A), TMT-B, clock drawing test (CDT) and semantic fluency test (SFT), whereas PD-D patients performed worse in most EF tests. With the deterioration of cognitive function, the concentration of serum GDNF and HVA in PD patients decreased. In the PD group, the serum GDNF and HVA levels were negatively correlated with TMT-A (*r*_GDNF_ = −0.304, *P* < 0.01; *r*_HVA_ = −0.334, *P* < 0.01) and TMT-B (*r*_GDNF_ = −0.329, *P* < 0.01; *r*_HVA_ = −0.323, *P* < 0.01) scores. Serum GDNF levels were positively correlated with auditory verbal learning test (AVLT-H) (*r* = 0.252, *P* < 0.05) and SFT (*r* = 0.275, *P* < 0.05) scores. Serum HVA levels showed a positively correlation with digit span test (DST) (*r* = 0.277, *P* < 0.01) scores. Stepwise linear regression analysis suggested that serum GDNF and HVA concentrations and UPDRS-III were the influence factors of TMT-A and TMT-B performances in PD patients.

**Conclusion:**

The decrease of serum GDNF concentration in PD patients was associated with impaired inhibitory control, cognitive flexibility, and attention performances. The changes of GDNF and HVA might synergistically participate in the occurrence and development of executive dysfunction in PD patients.

## Introduction

Mild cognitive impairment (MCI) is a transitional stage between normal aging and senile dementia, with a high risk of developing dementia (Svenningsson et al., 2012; Gonzalez-Redondo et al., 2014; Brennan et al., 2017). In patients with Parkinson’s disease (PD), the incidence of MCI can reach 40% even in the early stage of the disease course (Litvan et al., 2012; Gonzalez-Redondo et al., 2014). Executive dysfunction (EDF) is the most prominent feature of PD with cognitive dysfunction, affecting about 30% of all PD patients. This deficit can occur in the early course of PD, and includes impairments in the coordination of a range of cognitive process required to achieve complex, goal-oriented and new cognitive operations (Parker et al., 2013), manifesting as impairments in the three core executive functions (EFs) (working memory, cognitive flexibility, and inhibitory control), as well as in higher-level EFs such as reasoning, problem solving, and planning (Diamond, 2013).

In PD patients, the degeneration of frontal-striatal dopamine pathway contributes to EDF, manifesting as deficits in cognitive flexibility, planning, inhibitory control and working memory, usually relate to MCI in PD; whereas the degeneration of cholinergic systems and Lewy body pathological changes in the posterior brain system result in the visuospatial and memory dysfunction, relate to dementia of PD (Kehagia et al., 2010, 2013). A study suggested that homovanillic acid (HVA), as the main metabolite of dopamine, could be used as an intracerebral marker of dopamine function and metabolism (Saracino et al., 2006).

Glial cell line-derived neurotrophic factor (GDNF) has a strong neurotrophic effect on a variety of neurons, especially on dopaminergic neurons (DANs). GDNF promotes the survival, morphological differentiation, damage repair, and dopamine release of DANs, and regulates their excitability in the midbrain (Grondin et al., 2003; Wang et al., 2011; Brown et al., 2018), and play important role in motor symptoms of animal models of PD. So we wonder if serum GDNF may be involved in the EDF of PD patients, by acting alone or in conjunction with HVA (Liu et al., 2020).

To this end, we studied the correlation of the levels of GDNF and HVA in the peripheral blood serum of PD patients with EF and its components (working memory, cognitive flexibility, and inhibitory control), in order to explore the possible mechanism of GDNF participating in the occurrence and development of EDF, and to provide a theoretical basis for early identification of cognitive dysfunction in PD.

## Materials and methods

### Subjects

We enrolled a total of 105 PD outpatients and inpatients from the Neurology Department of the Affiliated Hospital of Xuzhou Medical University between May 2016 and October 2017. Inclusion criteria: (1) aged between 40 and 80 years old; (2) able to complete all cognitive tests under doctor’s instructions, and had no difficulties in listening, understanding and writing; (3) the diagnosis of PD was made independently by two experienced neurologists based on the UK PD Society Brain Bank Clinical Diagnostic Criteria (Hughes et al., 1993); (4) written informed consents were signed by the patients or their legal representatives. Exclusion criteria: (1) with neurological histories other than PD, e.g., moderate or serious brain injury, stroke, and vascular dementia confirmed by CT/MRI; (2) secondary parkinsonism induced by drugs, vascular lesions, tumors, trauma, and other insults, Parkinsonism-plus syndrome such as the progressive supranuclear palsy and multiple system atrophy; (3) major psychological diseases such as anxiety, depression and schizophrenia (Stuart et al., 2020); (4) systemic disease affecting the heart, liver or kidney, and other diseases that might affect cognitive function (Gonzalez-Redondo et al., 2014).

We also recruited 45 healthy normally aging volunteers with comparable age, sex, and education level with the PD patients as the healthy control (HC) group. Detailed materials and methods were as described in our previous research (Liu et al., 2020).

This study was approved by the ethics committee of the Affiliated Hospital of Xuzhou Medical University (approval no: XYFY2017-KL047-01). Written informed consents were signed by all subjects for enrollment.

### Neuropsychological assessment

We assessed the global cognitive function and the five cognitive domains (attention, executive function, language, memory, and visuospatial function) of all subjects. The global cognitive function was assessed using the mini-mental state examination (MMSE). Attention was tested by forward digit span test (DST). Language was assessed by Boston naming test (BNT). Memory was examined by auditory verbal learning test-HuaShan version (AVLT-H). Visuospatial function was evaluated by clock copying test (CCT).

Executive function was evaluated by trail making test (TMT) (inhibitory control), backward DST (working memory), clock drawing test (CDT) (planning and inhibitory control), semantic fluency test (SFT) (cognitive flexibility).

All patients were evaluated in the “ON” state, and those with scores 1.5 SD lower than the HC group were considered to have neuropsychological impairment. PD patients were classified using the diagnostic criteria recommended by Movement Disorder Society (MDS) of literature support as PD-MCI (Litvan et al., 2012) (*n* = 41) and PD-D (Emre et al., 2007) (*n* = 20). Those who did not fulfill criteria for PD-MCI or PD-D were classified as PD-N (*n* = 44).

### Blood sampling

We took the blood sample from overnight fasting subjects between 8:00 a.m. and 9:00 a.m., including both the PD group and HC group. Blood samples were centrifuged at 1,500 × *g* for 15 min, aliquoted, and stored at −80°C until further examination.

### Determination of serum GDNF and HVA concentrations

Serum GDNF and HVA concentrations were determined by enzyme linked immunosorbent assay (ELISA) and liquid chromatography tandem mass spectrometry (LC-ESI-MS/MS analysis), respectively.

### Statistical analysis

SPSS 16.0 software was used for statistical analysis. Normality test was performed and normally distributed data were shown as mean ± standard deviation (X ± SD), whereas the non-normal data were expressed as median (interquartile range). *T*-test was used for comparison between two groups. For multi-group comparison, parametric data were analyzed by analysis of variance (ANOVA), and non-parametric data were compared by Kruskal–Wallis test, with LSD method (or Bonferroni method) for multiple comparison between groups. Correlation analysis was carried out using Pearson or Spearman correlation coefficient according to the distribution of variables. Stepwise linear regression analysis was used to identify the influence factors of cognitive test performance. Chi-square test was performed on binary variables. A two-sided *P* < 0.05 was considered statistical significance.

## Results

### Comparison of the general demographic data between the PD and HC groups

A total of 105 PD patients (PD group) were included in the study and classified into three groups: PD patients with normal cognitive function (PD-N, *n* = 44), PD patients with MCI (PD-MCI, *n* = 41), PD patients with dementia (PD-D, *n* = 20), and were compared with healthy controls (HC, *n* = 45) for general demographic data as well as MMSE and GDS-30 scores (Table 1). There was no significant difference in the age, sex ratio and education level among all four groups (*P* > 0.05). Compared with the HC group, PD patients had worse MMSE scores and more severely depressive symptoms (MMSE: *H* = 81.192, *P* < 0.001; GDS-30 *F*_3,150_ = 9.958, *P* < 0.001).

**TABLE 1:** Comparison of the general demographic data between the PD and HC groups.

	PD-N (*n* = 44)	PD-MCI (*n* = 41)	PD-D (*n* = 20)	HC (*n* = 45)	*P*-value
Age (years)	61.66 ± 8.31	65.02 ± 8.61	67.75 ± 6.16	64.56 ± 8.08	0.056
Education (years)	10.82 ± 2.36	9.93 ± 2.30	9.35 ± 2.23	10.58 ± 2.96	0.090
Male *n* (%)	27 (61.4%)	23 (56.1%)	6 (30.0%)	27 (55.3%)	0.100
MMSE score	28.5 (27–29)	27 (27–28)	22 (20–23)	29 (28–29.5)	<0.001
GDS-30 score	9.48 ± 6.16	7.76 ± 4.6	15.15 ± 6.48	6.62 ± 3.97	<0.001
UPDRS-III score	20.24 ± 8.03	28.24 ± 8.88	34.08 ± 7.28	–	<0.001
Hoehn-Yahr grade	1.75 (1–2)	2.5 (2–2.75)	3 (2.5–3)	–	<0.001
Disease course (months)	24 (9–57)	24 (12–66)	54 (24–93)	–	0.013
LED (mg/d)	297.16 ± 27.48	354.57 ± 31.37	534.38 ± 56.19	–	<0.001

PD-N, PD with normal cognitive function; PD-MCI, PD with mild cognitive impairment; PD-D, PD with dementia; HC, normal control; MMSE, mini-mental state examination; GDS-30, Geriatric Depression Scale-30; UPDRS-III, unified PD rating scale part III exercise evaluation; LED, equivalent levodopa dose. Data were represented as X ± SD or median (interquartile range).

We also compared the clinical symptoms (UPDRS-III and Hoehn-Yahr grades), course of disease and equivalent levodopa dose (LED) among PD-N, PD-MCI and PD-D groups (Table 1), and found a decrease of cognitive function with the increase of UPDRS-III score (*H* = 21.852, *P* < 0.001), Hoehn-Yahr grade (*H* = 30.868, *P* < 0.001) and the progress of disease course (*H* = 8.655, *P* = 0.013) in PD patients. There was a significant difference in LED among the three PD groups (*F*_2,105_ = 9.386, *P* < 0.001). LED of the PD-D group was higher than that in the PD-N group and PD-MCI group (*P* < 0.05).

### Different degrees of EF impairment among the PD groups

For all the cognitive tests that we performed, there were statistically significant differences among groups (*P* < 0.001). The scores of the PD-D group were lower than those of the PD-N, PD-MCI and HC groups (*P* < 0.001), except for CDT (PD-MCI vs. PD-D, *P* = 0.008). The performances of the PD-MCI group were lower than those of the HC group (*P* < 0.001) and PD-N group (*P* < 0.001) in four EF tests TMT-A, TMT-B, CDT, and CFT. For backward DST: HC vs. PD-MCI, *P* = 0.011, PD-N vs. PD-MCI, *P* = 0.197. There was no significant difference in the rest of the tests among the PD groups or in all test between the PD-N and HC groups (*P* > 0.05) (Table 2).

**TABLE 2:** Comparison of neuropsychological test results between the PD groups and the HC group.

	PD-N (*n* = 44)	PD-MCI (*n* = 41)	PD-D (*n* = 20)	HC (*n* = 45)	*P*-value
Forward DST (n)	6 (6–8)	6 (5–7)	4 (3.5–4)	6 (6–7.5)	<0.001
Backward DST (*n*)	4 (3–5)	4 (3–4)	2 (2–3)	4 (4–5)	<0.001
Total digit span (*n*)	11 (9.5–12)	10 (9–11)	6 (6–7)	4 (4–5)	<0.001
TMT-A (s)	60.80 ± 16.31	89.07 ± 36.46	120.95 ± 41.09	55.42 ± 27.22	<0.001
TMT-B (s)	134.18 ± 29.09	205.29 ± 50.67	270.25 ± 29.08	132.84 ± 48.61	<0.001
CDT	4 (4–4)	3 (2–4)	1 (1–1.5)	4 (4–4)	<0.001
SFT (*n*)	15.36 ± 2.87	12.37 ± 2.87	10.75 ± 3.14	15.76 ± 2.87	<0.001
BNT (*n*)	22.55 ± 2.71	20.46 ± 3.39	18.60 ± 3.28	23.51 ± 3.56	<0.001
AVLT-H free recall (n)	17.95 ± 4.64	12.49 ± 4.67	8.10 ± 4.14	17.82 ± 5.32	<0.001
CCT	4 (4–4)	4 (3–4)	1 (1–4)	4 (4–4)	<0.001

PD-N, PD with normal cognitive function; PD-MCI, PD with mild cognitive impairment; PD-D, PD with dementia; HC, normal control; DST, digit span test; TMT, trail making test; CDT, clock drawing test; SFT, semantic fluency test; BNT, Boston naming test; AVLT-H, auditory verbal learning test-HuaShan version; CCT, clock copying test. Data were represented as X ± SD or median (interquartile range).

### The serum GDNF and HVA concentrations of the PD group were lower than the HC group

There was a significant difference in serum GDNF level between the PD group (475.65 ± 139.43 pg/mL) and the HC group (572.96 ± 210.55 pg/mL) (*t* = 2.706, *P* = 0.008). The serum GDNF concentration of the PD-N group (532.13 ± 138.30 pg/mL) was significantly higher than that of the PD-MCI group (439.87 ± 139.59 pg/mL, *P* < 0.01) and the PD-D group (424.73 ± 101.96 pg/mL, *P* < 0.01). There was no statistically significant difference in the serum GDNF level between the PD-MCI group and the PD-D group (*P* = 0.899).

**FIGURE 1:**
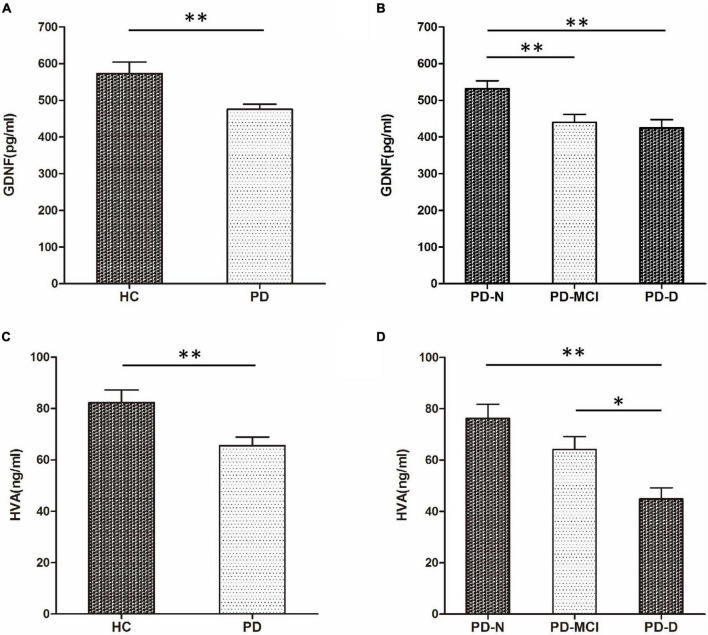
Comparison of serum glial cell line-derived neurotrophic factor (GDNF) and homovanillic acid (HVA) concentrations among groups. **(A)** Comparison of serum GDNF concentration between HC and PD groups. **(B)** Comparison of serum GDNF concentrations among PD-N, PD-MCI, and PD-D groups. **(C)** Comparison of serum HVA concentration between HC and PD groups. **(D)** Comparison of serum HVA concentration among PD-N, PD-MCI, and PD-D groups. *P < 0.05 and ^**^P < 0.01.

The serum HVA level in PD groups (65.55 ± 33.88 ng/mL) was significantly lower than the HC group (82.27 ± 32.98 ng/mL) (*t* = 2.972, *P* = 0.006). The serum HVA concentration of the PD-D group (44.88 ± 19.28 ng/mL) was lower than that of the PD-N group (76.24 ± 36.79 ng/mL, *P* = 0.001) and the PD-MCI group (64.16 ± 31.81 ng/mL, *P* = 0.035). There was no significant difference in serum HVA level between the PD-D group and the PD-MCI group (*P* = 0.087) **(Figure 1).**

### Serum GDNF level was related to TMT and SFT, and serum HVA level was related to TMT and DST in PD groups

The comparison of the correlation between GDNF serum concentration and neuropsychological test showed that in PD group, serum GDNF level was negatively correlated with TMT-A and TMT-B scores, and positively correlated with AVLT-H and SFT performances. There was no correlation with the HC groups.

**FIGURE 2:**
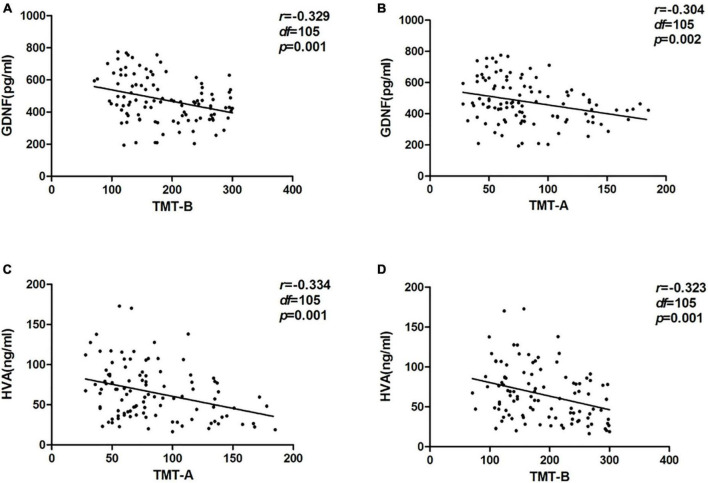
Correlation between serum glial cell line-derived neurotrophic factor (GDNF) and homovanillic acid (HVA) concentrations and trail making test (TMT) in the PD group. **(A)** Correlation between serum GDNF concentration and TMT-A. **(B)** Correlation between serum GDNF concentration and TMT-B. **(C)** Correlation between serum HVA concentration and TMT-A. **(D)** Correlation between serum HVA concentration and TMT-B.

Through correlation analysis of serum HVA concentration and cognition function, we found that in the PD groups, HVA serum level was negatively correlated with TMT-A and TMT-B scores, and positively correlated with backward DST and total DST scores. There was no correlation with the HC group (Table 3 and Figure 2).

**TABLE 3:** Relationship between serum glial cell line-derived neurotrophic factor (GDNF) or high vanillic acid (HVA) concentration and neuropsychological tests (correlation coefficient *r*).

	GDNF (pg/mL)		HVA (ng/mL)	
	PD (*n* = 105)	HC (*n* = 45)	PD (*n* = 105)	HC (*n* = 45)
Backward DST^a^	0.143	0.238	0.277**	0.246
Forward DST^a^	0.175	-0.015	0.185	0.049
Total DST^a^	0.186	0.132	0.25*	0.15
TMT-A (s)^b^	-0.304**	0.006	-0.334**	-0.165
TMT-B (s)^b^	-0.329**	-0.083	-0.323**	-0.165
SFT^b^	0.275*	0.164	0.104	0.213
CDT^a^	0.177	0.005	0.166	0.046
BNT^b^	0.061	0.014	0.006	0.256
AVLT-H^b^	0.252*	-0.153	0.147	0.051
CCT^a^	0.092	0.035	0.152	0.046

DST, digit span test; TMT, trail making test; SFT, semantic fluency test; CDT, clock drawing test; CCT, clock copying test; AVLT-H, auditory verbal learning test-HuaShan version.

^a^Spearman correlation coefficient.

^b^Pearson’s correlation coefficient.

*P < 0.05 and **P < 0.01.

### Serum GDNF and HVA concentrations and UPDRS-III were the influence factors of TMT-A and TMT-B performances in PD patients

The neuropsychological tests performed in this study were taken as dependent variables, and the GDNF, HVA, sex, age, course of disease, medication, UPDRS-III, Hoehn-Yahr grade were taken as independent variables for stepwise linear regression analysis, in order to identify the influence factors that predict the performance of neuropsychological tests. The results were as follows: When using TMT-A as the dependent variable (*F*_3,105_ = 25.756, *P* < 0.001, *R*^2^ = 0.665, adjusted *R*^2^ = 0.639), among the included variables GDNF, HVA and UPDRS-III had statistically significant impacts on the test results (*P* < 0.05) (Table 4). When using TMT-B as the dependent variable (*F*_4,105_ = 25.756, *P* < 0.001, *R*^2^ = 0.700, adjusted *R*^2^ = 0.668), among the included variables, GDNF, HVA, UPDRS-III, and age had statistically significant impacts on the test results (*P* < 0.05) (Table 5). The performances of TMT-A and TMT-B improved with the increase of GDNF and HVA concentrations, and deteriorated with the aggravation of motor symptoms. The performance of TMT-B also deteriorated with the increase of age.

**TABLE 4:** Stepwise linear regression analysis result of TMT-A as the dependent variable.

Independent variable	Unstandardized coefficients (B)	Standard error (SE)	Standardized regression coefficient (β)	*T*-value	*P*-value
Constant term	172.262	18.699	−	9.201	<0.001
GDNF	-0.107	0.030	-0.356	-3.582	0.001
HVA	-0.387	0.101	-0.415	-3.846	<0.001
UPDRS-III	1.203	0.378	0.325	3.185	0.003

GDNF, glial cell line-derived neurotrophic factor; HVA, homovanillic acid; UPDRS-III, unified PD rating scale part III-motor function.

**TABLE 5:** Stepwise linear regression analysis results of TMT-B test as dependent variable.

Independent variable	Unstandardized coefficients (B)	Standard error (SE)	Standardized regression coefficient (β)	*T*-value	*P*-value
Constant term	162.492	32.873	−	4.943	<0.001
GDNF	-0.119	0.036	-0.272	-3.284	0.002
HVA	-0.454	0.129	-0.321	-3.506	0.001
UPDRS-III	1.829	0.448	0.395	4.088	<0.001
Age	1.431	0.439	0.294	3.259	0.002

GDNF, glial cell line-derived neurotrophic factor; HVA, homovanillic acid; UPDRS-III, unified PD rating scale part III-motor function.

When using SFT was taken as the dependent variable, the influences of UPDRS-III and education level on the test performance were statistically significant. The SFT performance worsened with the aggravation of motor symptoms and progression of disease, and improved with the increase of education level. Hoehn-Yahr grade was an independent influence factor of CDT, AVLT, backward DST, and total DST, which increased with the severity of the disease. The influence factor of BNT performance was UPDRS-III, which the test performance deteriorated with the aggravation of motor symptoms.

## Discussion

The cognitive progress of PD patients can develop from the normal cognitive function stage (PD with normal cognition, PD-N) to the transition stage of PD with MCI (PD-MCI), and finally deteriorates to dementia (PD with dementia, PD-D) (Litvan et al., 2012). There is a high risk of MCI developing dementia (Svenningsson et al., 2012). Cognitive impairment in PD affects patient’s EF, attention, memory, language and visuospatial function, among which the EDF seems to be the most serious (Kehagia et al., 2013). In PD patients, the depletion of dopamine may lead to the disruption of the frontal-striatal circuit, which contributes to EDF (Uekermann et al., 2004; Kudlicka et al., 2011; Zhang et al., 2020).

In this study, the PD-N patients did not show obvious EDF. PD-MCI patients mainly suffered from impairment of attention, planning, inhibitory control, attention switching, and cognitive flexibility, which largely accorded with the results of Kudlicka et al. (2011), that is, impairment of EFs including the cognitive flexibility, reaction inhibition, inhibitory distraction of attention, and attention maintenance can occur in the early stage of PD. Unsurprisingly, PD-D patients performed worse in extensive EF tests. At present, the exact development pattern of EDF in PD is still controversial (Diamond, 2013), which may be attributed to the complex pathoanatomical mechanism of PD. The dual syndrome hypothesis holds that degeneration of frontal-striatal dopamine pathway contributes to executive dysfunction, manifesting as deficits in cognitive flexibility, planning, inhibitory control and working memory, usually relate to MCI in PD; whereas the degeneration of cholinergic systems and Lewy body pathological changes in the posterior brain system result in the visuospatial and memory dysfunction, relate to dementia of PD (Kehagia et al., 2010, 2013). The dopamine overdose hypothesis holds that dopamine deficiency first appears in the dorsal striatum at the early stage of PD, whereas dopamine is relatively better preserved in the ventral striatum, and the dorsolateral frontal striatal pathway is more likely to degenerate in the early stage of PD than the ventral frontal striatal pathway. Therefore, impairment of attention switching mainly occurs in the early stage, whereas the reverse learning ability (cognitive flexibility) is better preserved in the dorsal side (Vaillancourt et al., 2013). The results of this study found that the impaired performance of attention switching and cognitive flexibility in early PD patients might be related to the overdose effect of dopamine medication on the ventral striatum. On the other hand, it might also be association with the complex and vague features of EF *per se* (Zhang et al., 2020).

This study also found lower peripheral serum GDNF level in the PD patients than the health controls, which was consistent with the results of previous clinical studies. One study found that the GDNF concentration in cerebrospinal fluid of PD patients was lower than that of normal controls, and was lower in early PD patients than late PD patients (Vaillancourt et al., 2013). Nagatsu and Sawada (2007) reported that the concentration of neurotrophic factors including GDNF decreased in the substantia nigra of PD patients. A large number of trials had used GDNF as a potential therapy to treat motor symptoms of PD. At present, gene and cell therapies are widely applied in preclinical and clinical researches, and have achieved certain success (Garbayo et al., 2016). However, the research of GDNF application in the field of cognition in PD patients was mostly limited to animal experiments. Some studies found that after intracerebroventricular injection of lentiviral vector carrying the human *GDNF* coding sequence in PD rats, the expression of GDNF increased and the spatial learning and memory ability improved (Lu-Nguyen et al., 2014). Another study described reduced GDNF concentration in the brain of rats with induced nerve infection, and their cognitive function, especially learning and memory, also deteriorated (Gui et al., 2017). It was also found that after amantadine treatment, the expression of GDNF in the hippocampus of PD rats increased, and their learning and memory abilities also improved (Zhang et al., 2014).

Through further exploration of the correlation between serum GDNF and neuropsychological test performance, this study found that the increase of GDNF concentration was correlated with better working memory, attention, inhibitory control, cognitive flexibility and memory. The correlation between serum GDNF concentration and EF tests has been reported previously, and it was found that the serum GDNF concentration of schizophrenic patients who were initially untreated was negatively correlated with the performance of TMT-B (Xiao et al., 2017). Niitsu et al. (2014) found that the GDNF concentration in the peripheral serum was positively correlated with DST performance in the healthy normal population. The research of Wang et al. (2011) showed that the plasma GDNF concentration of late-onset depression patients was positively correlated with the performance of backward DST and negatively correlated with that of TMT-B. These results show that with the increase of GDNF concentration, the test performance of attention and working memory improves, which is consistent with our results. We also found that GDNF is correlated with the performance of AVLT (memory), TMT-A (attention) and SFT (cognitive flexibility). We believed that the improvement of cognition induced by GDNF might be related to its strong neurotrophic and neuroprotective effects not only on DANs, but also on the serotonin neurons, norepinephrine neurons, cholinergic neurons. Studies have found that the complex neurotransmitter circuits are involved in various cognitive processes (Murley and Rowe, 2018; Hirano, 2021). For example, the dopamine system is involved in the regulation of EF by the frontal system (Halliday et al., 2014), the serotonin system is closely related to cognitive flexibility (Clarke et al., 2004), the norepinephrine system can affect set-switching, attention, response inhibition and reverse learning (Logue and Gould, 2014), and episodic memory coding and spatial memory processing are thought to rely on the acetylcholine system to maintain normal functions (Newman et al., 2012). GDNF might synergistically participate in the process of cognitive function through its neuroprotective effects by regulating the survival of these neurons and synaptic plasticity. Another theory believes that GDNF can antagonize the degeneration of neurons and thus delay the occurrence of cognitive decline. The specific mechanism is not completely clear, which may involve the antagonism of GDNF against oxidative stress, inhibition of microglial excitability and neuroinflammation (Gill et al., 2003; Tome et al., 2017).

In addition, this study also found that the serum HVA concentration in PD patients was lower than that in normal controls. HVA is the main metabolite of dopamine, and the measurement of HVA concentration in body fluid may be the most direct method to reflect the function and activity changes of human central DANs (Pickar et al., 1988). Through further analysis of the correlations between HVA concentration and the performances of EF tests in PD patients, we found that serum HVA level was closely related to the performance of backward DST, total DST and TWT in PD patient, suggesting the correlation between serum HVA level with the EF components such as working memory, inhibitory control, cognitive flexibility, and attention. This was consistent with the results of a previous study that the dopamine system is involved in the regulation of EF by the prefrontal cortex (Martinez et al., 2016).

The levels of GDNF and HVA in peripheral serum were negatively correlated with the performance of TMT-A and TMT-B in PD patients. Regression analysis showed that both GDNF and HVA were influence factors for TMT performance. A meta-analysis by Yuan and Raz (2014) showed that the prefrontal cortex (especially the lateral prefrontal cortex) was involved in the regulation of functional tests such as trial making tests. In this study, we proposed that there were two possible causes of EF impairment in PD patients. On one hand, primary PD is usually accompanied by EDF similar to that in patients with frontal lobe injury (Christopher and Strafella, 2013; Mak et al., 2014), especially the degeneration of the dorsolateral prefrontal cortex and its closely related dorsal striatum dopamine system (O’Callaghan et al., 2014; Koshimori et al., 2015). On the other hand, we suspected that GDNF might be involved in the development of EDF possibly through the following mechanisms: (1) GDNF indirectly affect EF through its protective and repair effects on DANs. GDNF has a protective effect on midbrain DANs. GDNF prevents the loss of DANs and the degeneration of dopaminergic nerve terminals, and increase the level of dopamine in the striatum (Olanow et al., 2015). (2) GDNF antagonizes aging-related cognitive decline through an unknown mechanism. It antagonizes neuronal degeneration through antioxidant stress, inhibition of microglial excitability, and neuroinflammation (Pertusa et al., 2008; Budni et al., 2015). (3) More consistent evidence proved that brain-derived neurotrophic factor and GDNF participate in the pathophysiological process of PD (Nagatsu and Sawada, 2007). Therefore, GDNF might affect the cognitive process of PD patients through a yet unknown mechanism.

## Conclusion and expectation

Under our experimental conditions, this study found that the decrease of serum GDNF concentration in PD patients was associated with inhibitory control, cognitive flexibility, and attention performances. The changes of GDNF and HVA might synergistically participate in the occurrence and development of executive dysfunction in PD patients. Of course, due to the limitation of funds and other conditions, our research also has some shortcomings. We did not use objective cognitive assessment tools, such as functional MRI or PET scanning, to evaluate EF. Our study was a cross-sectional study rather than a cohort study and without follow-up study. All subjects were limited to the affiliated hospital of Xuzhou Medical University, and no other multicenter study data. In the future, more in-depth studies will be carried out combining animal models, cell experiments, functional imaging methodologies and multicenter prospective cohort study to explore its possible mechanism. Although there are a few limitations in this study, we have solved some problems in this field. All in all, GDNF might be used as a biomarker to identify early cognitive decline in PD, and might serve in clinical practice.

## Data availability statement

The original contributions presented in this study are included in the article/supplementary material, further inquiries can be directed to the corresponding authors.

## Ethics statement

This study was approved by the Ethics Committee of the Affiliated Hospital of Xuzhou Medical University (approval no: XYFY2017-KL047-01). Written informed consents were signed by all subjects for enrollment. The patients/participants provided their written informed consent to participate in this study.

## Author contributions

CR and D-SG: conception, design, and administrative support. S-YT, R-WW, and QL: provision of study materials. S-YT, R-WW, QL, YL, X-YY, and D-QG: collection and assembly of data. S-YT, D-SG, and CR: data analysis and interpretation. All authors manuscript writing and final approval of manuscript.
